# Incentives and barriers to HIV testing among female sex workers in Ceará

**DOI:** 10.11606/S1518-8787.2018052000300

**Published:** 2018-06-21

**Authors:** Telma Alves Martins, Ligia Kerr, Raimunda Hermelinda Maia Macena, Rosa Salani Mota, Inês Dourado, Ana Maria de Brito, Laetitia Atlani_Dualt, Laurent Vidal, Carl Kendall

**Affiliations:** I Secretaria de Saúde do Estado do Ceará. Coordenadoria de Promoção e Proteção à Saúde. Núcleo de Vigilância Epidemiológica. Fortaleza, CE, Brasil; II Universidade Federal do Ceará. Faculdade de Medicina. Departamento de Saúde Comunitária. Fortaleza, CE, Brasil; III Universidade Federal do Ceará. Faculdade de Medicina. Curso de Fisioterapia. Fortaleza, CE, Brasil; IV Universidade Federal do Ceará. Departamento de Estatística e Matemática Aplicada. Fortaleza, CE, Brasil; V Universidade Federal da Bahia. Instituto de Saúde Coletiva. Salvador, BA, Brasil; VI Fundação Oswaldo Cruz. Instituto Aggeu Magalhães. Recife. PE, Brasil; VII Institut de Recherche pour le Développement. Marseille, França; VIII Tulane School of Public Health and Tropical Medicine. Global Community Health and Behavioral Sciences. New Orleans, United States

**Keywords:** Sex Workers, HIV Seroprevalence, Health Knowledge, Attitudes, Practice, Unsafe Sex, prevention & control, Risk Factors, HIV Infections, prevention & control, Profissionais do Sexo, Soroprevalência de HIV, Conhecimentos, Atitudes e Prática em Saúde, Sexo sem Proteção, prevenção & controle, Fatores de Risco, Infecções por HIV, prevenção & controle

## Abstract

**OBJECTIVE:**

Estimating HIV prevalence and describing the incentives and barriers for HIV testing among female sex workers.

**METHODS:**

This cross-sectional study recruited 402 women aged 18 years or older, residing in Fortaleza, state of Ceará, Brazil, who reported having had sexual intercourse in exchange for money in last four months. The sample was recruited using Respondent Driven Sampling, between August and November 2010.

**RESULTS:**

The 84.1% of the sample tested and the estimated prevalence of HIV infection was 3.8%. The sample was young (25 to 39 years ), single (80.0%), with one to three children (83.6%), had eight or more years of schooling (65.7%), and belonged to social classes D/E (53.1%). The majority worked in fixed locations (bars, motels, hotels, sauna - 88.9%), and prostitution was their only source of income (54.1%). About 25% of the sample did not know where to test in the public health sector and 51.8% either never tested or hadn’t tested for over a year or more. The main reported barriers to testing were the perceptions that there was no risk of becoming infected (24.1%), and, alternatively, fear of discrimination if the test was positive (20.5%). Incentives for testing were the greater availability of testing sites (57.0%) and health facilities with alternative schedules (44.2%).

**CONCLUSIONS:**

Prevalence for HIV was similar to that found in other Brazilian cities in different regions of the country, although higher than the general female population. Non-traditional venues not associated with the health system and availability of testing in health units during non-commercial hours are factors that encourage testing. Not considering oneself to be at risk, fear of being discriminated against and not knowing testing locations are barriers.

## RESUMO

**OBJETIVO:**

Estimar a prevalência do HIV e descrever os incentivos e barreiras à realização do teste para o HIV entre mulheres profissionais do sexo.

**MÉTODOS:**

Este estudo transversal recrutou 402 mulheres de 18 anos ou mais, residentes em Fortaleza, CE, que informaram ter tido relação sexual em troca de dinheiro nos últimos quatro meses. A amostra foi recrutada por meio da técnica *Respondent Driven Sampling,* entre agosto e novembro de 2010.

**RESULTADOS:**

A adesão ao teste de HIV foi de 84,1% e a prevalência estimada da infecção pelo HIV foi de 3,8%. A amostra era jovem (25 a 39 anos), solteira (80,0%), com um a três filhos (83,6 %), tinham oito anos ou mais de estudo (65,7%) e pertencia às classes sociais D/E (53,1%). A maioria exercia a profissão em locais fechados (bares, motéis, hotéis, sauna – 88,9%), e a prostituição era a única fonte de renda (54,1%). Cerca de 25% da amostra desconhecia onde o teste de HIV era realizado na rede pública e 51,8% nunca fez o teste ou se testou há um ano ou mais. As principais barreiras ao teste foram acreditar que não corre risco de se infectar (24,1%) e o medo da discriminação caso o teste fosse reagente (20,5%). Os incentivos foram relacionados à maior oferta de locais para o teste (57,0%) e de unidades de saúde com horários alternativos (44,2%).

**CONCLUSÕES:**

A prevalência foi semelhante à encontrada em outras cidades brasileiras de diferentes regiões do país, apesar de superiores a de mulheres não profissionais do sexo. A disponibilidade do teste em locais não relacionados à saúde e a oferta nas unidades básicas em horário não comercial são fatores que incentivam a realização do teste. Não se considerar sob-risco, medo de ser discriminada e desconhecimento dos locais onde o teste é realizado podem ser uma barreira para a realização do exame.

## INTRODUCTION

In many countries, especially in low- and middle-income countries, female sex workers (FSW) have higher levels of HIV infection than women of reproductive age who do not engage in sex work (FNSW). A study carried out in 10 Brazilian capitals showed that the prevalence of HIV among FSW may be up to 15 times higher than in women in general^1^ ^,^ ^2^.

The risk of acquiring HIV infection is influenced by FSW behavioral, biological and work context factors. Behaviors such as injecting drug use, early sexual initiation, multiple sexual partnerships and inconsistent use of condoms lead to an increased risk of acquiring HIV and other sexually transmitted diseases, such as syphilis and viral hepatitis. Structural factors such as violation of rights and violence (physical and sexual) contribute to the increased risk of HIV and the vulnerability of FSW^2^ ^,^ ^4^.

The scenario of stigma, discrimination and social violence favors denial of sex work and amplifies vulnerabilities. Social exclusion contributes to keeping FSW from health services. This increases the risks to which they are subjected in the practice of the profession, such as sexually transmitted infections, intersection of injecting drug use, sex with more HIV-positive partners, low and inconsistent use of the condom, among others^1–3,5,6^. The combination of all these factors together with the limitation of financial accessibility, illiteracy, discrimination, social exclusion, and segregation make it a challenge to prevent and treat HIV infection and other sexually transmitted diseases among FSW^1^ ^,^ ^4^.

Early diagnosis and treatment of HIV infection contribute to reducing the clinical progression of the disease and transmission of the virus to the partner(s)^7^ ^,^ ^8^. Thus, HIV testing is strongly recommended for populations at frequent risk of acquiring the infection, including FSW, and is recommended to be performed at intervals of three to four months^5^. Despite recognizing FSW as the key population to be prioritized for the control of the AIDS epidemic, the incentives and barriers to conducting HIV testing among FSW are poorly studied^1^ ^,^ ^3^.

This article aimed to describe the main incentives and barriers to conducting HIV testing among FSW. The findings will provide subsidies to health managers facing challenges to reduce HIV transmission rates among key populations in the country.

## METHODS

This is a cross-sectional study conducted in Fortaleza, state of Ceará, Brazil, between August and November 2010, with 402 women aged 18 years or older who reported sexual intercourse in exchange for money in the last four months. The FSW were recruited using the Respondent Driven Sampling (RDS) method. RDS was proposed in the late 1990s and is used to access hard-to-reach populations, including FSW because they have stigmatized and even illegal behavior in some countries^9^ ^,^ ^10^.

The formative research was carried out with researchers and leaders of the FSW movement to present the study, to negotiate participation, and to define the strategies for fieldwork, such as the selection of the seeds (the first recruiters), the nature and size of reimbursement, and other procedures (locations, working days, daily schedule), as well as refine inclusion criteria.

The sample was calculated using an HIV prevalence estimate of 6.1% (3.7% of sampling error, α = 5.0% and power of 70.0%), an official figure for the country^11^. The minimum sample size was 204 women. Salganik^12^ reviews design effects (deff) for RDS of 2–6. We used a deff of two to compensate for variance from simple random sampling introduced by the method. Thus, the final sample consisted of 410 women, including five seeds. Of the 410 FSW recruited, six (1.5%) were ineligible and two (0.5%) did not complete the questionnaire; thus, 402 were included in the final sample.

Seeds were recruited purposively, taking into consideration the diversity of income, schooling, and sex work sites (downtown and the coastal area). Two NGO (non-governmental organizations) directed to FSW assisted in the selection of the seeds. Each seed received three numbered and non-reproducible coupons to recruit FSW from their social networks. The procedure was repeated until the sample size was reached.

Each woman interviewed received a primary incentive (R$15.00) for reimbursement of expenses (transportation and food) and a secondary incentive (R$10.00) for each recruited FSW that participated in the study.

Data collection was conducted in two public health facilities (A: daytime and B: afternoon and evening hours). The interviews could be pre-scheduled by phone or spontaneously in the health unit. The data were collected using computer assisted personal interview (CAPI) on a Pocket PC^®^, and with counseling and rapid test (RT) for HIV. The questionnaire included sections for social network, sociodemographic profile, history of HIV testing, sexual behavior, knowledge about HIV/AIDS, health care and STD; discrimination and violence.

Counseling and the RT for HIV were offered to all FSW after the questionnaire was completed. If the result was positive, the woman was referred to one of the Specialized Ambulatory Services (SAE) that compose the care network for persons living with HIV/AIDS (PVHA) in Fortaleza.

The variables were examined for missing and extreme values as well as for logical consistency. Next, a descriptive analysis was conducted and gross and adjusted prevalence of the variables of greatest interest with 95% confidence intervals (CI) were identified to compare with national studies. In this phase of the analysis, Respondent Driven Sampling Analysis Tool (RDSat^®^), version 6.0 was used. We used parameters of 15,000 resampling and confidence intervals equal to 95% (α = 0.025).

The study was approved by the Research Ethics Committee of the Faculdade de Medicina da Universidade Federal do Ceará (Protocol 263/09). All participants signed the informed consent form.

## RESULTS

Almost half of the interviewees were young (between 25 and 39 years of age, mean = 33.0 years, SD = 10.8 years, range = 18–67 years). The majority identified as color racial category black/brown (81.9%), had eight or more years of schooling (65.7%), were single (80%), had one to three children (83.6%) and belonged to social classes D/E (53.1%). Most worked in fixed locations such as bars, motels, hotels, baths, and saunas (88.9%) and reported prostitution as their only work (54.1%) (Table 1).

**Table 1:** Sociodemographic characteristics of female sex workers. Fortaleza, state of Ceará, Brazil, 2010.

Sociodemographic characteristics	% (95%CI)*
Age group (years) (n = 402)	
≤ 24	30.3 (23.9–36.0)
25 to 39	45.5 (40.6–52.1)
≥ 40	24.2 (18.9–29.1)
Race/Color (n = 401)	
Non-black/Brown	18.1 (13.3–22.6)
Black/Brown	81.9 (77.4–86.7)
Education (years) (n = 226)	
1 to 3	9.9 (6.7–14.0)
4 to 7	24.4 (20.1–29.4)
≥ 8	65.7 (59.7–70.5)
Religion (n = 402)	
No religion/Other religion	29.2 (23.3–34.7)
Catholic	64.8 (59.1–70.8)
Evangelic	6.0 (3.6–8.8)
Marital status (n = 402)	
Never been married (single)	80.0 (75.3–84.3)
Married or lives with partner	7.7 (4.7–10.9)
Separate or Divorced/Widowed	12.3 (9.0–16.2)
Number of children (n = 333)	
1 to 3	83.6 (79.0–88.8)
4 or more	16.4 (11.2–21.0)
Monthly income in minimum wage - MW (n = 400)	
< 1	49.4 (43.4–56.3)
1 to 4	42.2 (35.9–48.3)
≥ 4	8.3 (4.7–12.1)
Social class (n = 401)	
B/C	46.9 (40.5–53.9)
D/E	53.1 (46.1–59.5)
Has worked as a FSW in another city (n = 401)	38.5 (32.6–44.4)
Place where she worked (n = 396)	
Streets and squares	31.3 (25.6–37.6)
Motel/Hotel/Spa and saunas	44.1 (37.3–51.6)
Nightclubs/Bars	44.8 (38.7–51.2)
Another locations	7.5 (4.8–11.6)
How much she charged (R$) (n = 402)	
1.00 to 29.00	49.5 (39.7–57.3)
30.00 to 49.00	16.4 (12.3–21.6)
50.00 to 99.00	20.5 (15.6–26.6)
100.00 or higher	13.6 (9.0–19.5)
Does other work besides prostitution (n = 400)	45.9 (39.0–51.4)

FSW: female sex workers

* Estimates adjusted by RDSat.

Almost all FSW reported knowing about the purpose and advantages of HIV testing (> 90%). However, high percentages of FSW did not report that testing would increase their survival or quality of life (74.1%); the importance of initiating treatment once infected by HIV (59.2%); or that testing would help them take better care of their health (67.0%). They did not report that one purpose of HIV testing was to protect their sexual partners (79.1%) or that testing should be done routinely for all (77.0%) (Table 2).

**Table 2:** Knowledge about the benefits of HIV testing for female sex workers. Fortaleza, state of Ceará, Brazil, 2010.

Knowledge of the benefits of HIV testing	% (95%CI)*
There is an advantage to HIV testing	93.8 (90.5–96.4)
The test detects the virus/HIV antibody	91.2 (87.5–94.7)
The test should be compulsory for all people	43.1 (37.9–48.4)
The test helps to start treatment soon if infected	40.8 (34.7–47.5)
The test is to take better care of the body and health	33.0 (27.7–39.0)
The test prevents disease and/or increases survival	25.1 (19.9–0.2)
The test should be done routinely for all	23.0 (19.1–27.9)
The test protects partners	20.9 (17.3–25.1)
The test helps to have sex without fear and/or without condoms	18.5 (14.8–22.9)

n = 402 for all variables.

* Estimates adjusted by RDSat.

About 25% of the sample did not know where to test in the public health sector. The 28.2% reported never testing for HIV, and 23.6% reported being tested more than a year previously or did not know when they were tested. Among those that who reported being tested, the Testing and Counseling Center (CTA) was the primary location (52.1%) and the rapid test was the method of choice (54.6%). The HIV testing in the survey was high (84.1%) and the estimated prevalence of HIV infection was 3.8% (Table 3).

**Table 3:** Knowledge of sex workers about HIV testing. Fortaleza, state of Ceará, Brazil, 2010.

HIV testing	% (95%CI)*
Knows where HIV testing is done for free (n = 402)	75.8 (91.2–81.3)
Has ever tested for HIV (n = 402)	70.0 (65.1–75.0)
Time since the last HIV test (n = 402)	
Less than 3 months ago	12.5 (8.4–18.4)
Over 3 months and less than 6 months	24.5 (19.2–30.7)
Between 6 months and 12 months	11.2 (7.8–15.7)
More than 1 year or does not know	23.6 (18.6–29.5)
Was never tested	28.2 (23.2–33.8)
Location of the last HIV test (n = 268)	
Counseling and Testing Center (CTA)	52.1 (44.1–61.9)
Health center	21.1 (13.6–27.7)
Public hospital	23.1 (16.4–29.9)
Private hospitals/laboratories	1.9 (0.5–3.5)
Other	1.8 (0.2–3.8)
Reported having already had a rapid HIV test (n = 268)	54.6 (46.4–63.3)
Tested in the study (n = 402)	84.1 (79.3–88.3)
Results of the HIV test in the study (n = 338)	
Positive	3.8 (1.2–6.9)
Negative	96.2 (93.1–98.8)

* Estimates adjusted by RDSat.

The main reported barriers to testing were the perceptions that there was no risk of becoming infected (24.1%), and, alternatively, fear of discrimination if the test was positive (20.5%). Incentives for testing were the greater availability of testing sites (57.0%) and health facilities with alternative schedules (44.2%) (Table 4).

**Table 4:** Incentives and barriers to HIV testing among female sex workers. Fortaleza, state of Ceará, Brazil, 2010.

HIV testing	% (95%CI)*
Reason for not having been tested for HIV (n = 128)	
Believes she is not at risk and/or does not belong to a risk group/believes that there was no risk	24.1 (8.6–46.7)
Fear of discrimination if positive	20.5 (8.4–26.6)
Do not know where the test is conducted	15.4 (3.4–31.0)
Believes in partner’s fidelity	15.1 (4.8–25.2)
Fear of breach of confidentiality in health unit	1.9 (- – -)
No time to go to the unit	1.6 (1.3-9.4)
Incentive to test in the study	
Available in places other than the health unit	57.0 (50.8–62.8)
Available at most units and/or after-hours	44.2 (38.7–50.6)
Knowing that diagnosis improves the quality of life/increases survival	24.6 (20.1–29.8)
Knowing that she will have access to AIDS medication and/or support from the Health Services	21.1 (16.7–25.8)
If it was the rapid or “saliva” test	21.8 (17.0–27.0)

UBS: Basic health unit

* Estimates adjusted by RDSat.

## DISCUSSION

Prevalence for HIV was similar to that found in other Brazilian cities in different regions of the country, although higher than the general female population. Non-traditional venues not associated with the health system and availability of testing in health units during non-commercial hours are factors that encourage testing. Not considering oneself to be at risk, fear of being discriminated against and not knowing testing locations are barriers.

The estimated prevalence of HIV infection in this study (3.8%, 95%CI 1.2–6.9) is similar to studies conducted in different regions of the country in the last 17 years^1^ ^,^ ^2^ ^,^ ^13^. Some factors need to be considered in interpreting HIV prevalence among FSW over the years: (i) most studies, including this one, were conducted in urban and more industrialized areas^1^ ^,^ ^2^ ^,^ ^14^; (ii) there is methodological diversity among the studies^2^ ^,^ ^13^ ^,^ ^14^; (iii) there were important political and social changes during this period that especially affected locations for recruitment of samples^2^ ^,^ ^4^ ^,^ ^8^ ^,^ ^15^ ^,^ ^16^.

Despite the recommendation for more frequent HIV testing among key populations^4^ ^,^ ^15^, the proportion of FSW that have been tested in the three months prior to the survey was low. Women in general, sex workers or not, associate HIV testing with prenatal care, rather than their exposure to risk^5^ ^,^ ^17^. Despite this, the proportion of the interviewees who had already been tested in life in this study is higher than in the general population of Brazil^2^ ^,^ ^17^.

The FSW showed a high level of knowledge about the purpose and benefits of the test, but few recognized the need for routine testing, the potential health benefits of testing, and the need to protect their sexual partners. Studies have reported an increase in FSW testing when self-perceived risks associated with sex work increase, such as denial of condoms by clients, forced sex due to lack of condoms, condom failure, use of psychoactive substances, among others^1^ ^,^ ^3^ ^,^ ^6^ ^,^ ^18^ ^-^ ^20^.

Lifestyle, socioeconomic factors and access to health services and persuasive health education can influence the perception of individual risk and the adoption of safe sex practices with clients or non-clients^2^ ^,^ ^5^ ^,^ ^21^. In this study, denial of the risk of becoming infected with HIV was the main barrier related to HIV testing. However, as reported elsewhere, unprotected sex with regular partners was more frequent than with commercial partners^5^ ^,^ ^22^.

The main reported incentives for HIV testing were the availability of testing at locations other than the public health facilities, or at off-hours, and to offer rapid testing. As other studies have pointed out these factors are associated with a reduction in the time spent testing^14^ ^,^ ^23^ ^,^ ^24^ and awaiting results^15^ ^,^ ^16^ and reduction in the fear of being recognized by accessing an HIV-related health service.

A significant proportion of FSW were unaware of the locations to test for free in the public health network, and this factor was identified as a barrier to testing^1^ ^,^ ^2^ ^,^ ^23^. The high levels of testing during the implementation of this study allows us to speculate that ease of access is one of the factors that can increase HIV testing^2^ ^,^ ^14^ ^,^ ^24^. However, access alone does not guarantee testing, since testing may be influenced by the perceived difficulty in coping with the diagnosis of infection^14^ ^,^ ^24^.

The fear of being discriminated against if the test is HIV-positive is also an important barrier to testing^1^ ^,^ ^2^ ^,^ ^5^ ^,^ ^16^. Situations of stigma and social exclusion resulting from socioeconomic status, profession, race, among others, seem to contribute to distancing FSW from the health system, reducing the chance of access to early diagnosis for HIV^2^ ^,^ ^5^ ^,^ ^6^ ^,^ ^24^.

This study had difficulties accessing FSW in social networks with higher income or schooling. The limitation may be associated with: (1) FSW with higher purchasing power have greater independence in the organization of their sex work and have low-density social networks; (2) organizing prostitution and pimping are considered crimes in the country, thus, for a number of reasons, owners of high-level houses of prostitution may be less inclined to participate in the study; and (3) sex work practices in the city are both widely geographically spread, and organized differently by class, so that social networks of FSW might not cross. The possible impact in this study of the lower participation of this higher status FSW group means that our prevalence may be higher, considering that these FSW are more selective in their clientele, can demand condom use and have broader access to health care.

The prevalence of HIV among FSW in Ceará is similar to that reported in other Brazilian cities in different regions of the country, although it is higher than that of the general female population. Structural conditions for access to HIV testing (availability of testing in non-health settings and out-of-hours services) are important elements in encouraging FSW testing. On the other hand, personal factors (denial of risk, fear of being discriminated against) and access to health services (lack of knowledge of where to test) may be barriers to HIV testing in this population.

Continued monitoring of HIV infection is imperative through behavioral surveillance, the development of research (including rural areas), and the design of new strategies to increase access to HIV testing. In addition, prevention actions involving civil society working with this public are fundamental.

## References

[1] Malta M, Magnanini MM, Mello MB, Pascom AR, Linhares Y, Bastos FI (2010). HIV prevalence among female sex workers, drug users and men who have sex with men in Brazil: a systematic review and meta-analysis. BMC Public Health.

[2] Damacena GN, Szwarcwald CL, Souza PR, Dourado I (2011). Risk factors associated with HIV prevalence among female sex workers in 10 Brazilian cities. J Acquir Immune Defic Syndr.

[3] Baral S, Beyrer C, Muessig K, Poteat T, Wirtz AL, Decker MR (2012). Burden of HIV among female sex workers in low-income and middle-income countries: a systematic review and meta-analysis. Lancet Infect Dis.

[4] Beyrer C, Crago AL, Bekker LG, Butler J, Shannon K, Kerrigan D (2015). An action agenda for HIV and sex workers. Lancet.

[5] Kakchapati S, Singh DR, Rawal BB, Lim A (2017). Sexual risk behaviors, HIV, and syphilis among female sex workers in Nepal. HIV AIDS (Auckl).

[6] Wahed T, Alam A, Sultana S, Alam N, Somrongthong R (2017). Sexual and reproductive health behaviors of female sex workers in Dhaka, Bangladesh. PLoS One.

[7] Marks G, Crepaz N, Senterfitt JW, Janssen RS (2005). Meta-analysis of high-risk sexual behavior in persons aware and unaware they are infected with HIV in the United States: implications for HIV prevention programs. J Acquir Immune Defic Syndr.

[8] Mahoney MR, Fogler J, Weber S, Goldschmidt RH (2009). Applying HIV testing guidelines in clinical practice. Am Fam Physician.

[9] Kendall C, Kerr LR, Gondim RC, Werneck GL, Macena RH, Pontes MK (2008). An empirical comparison of respondent-driven sampling, time location sampling, and snowball sampling for behavioral surveillance in men who have sex with men, Fortaleza, Brazil. AIDS Behav.

[10] Johnston LG, Malekinejad M, Kendall C, Iuppa IM, Rutherford GW (2008). Implementation challenges to using respondent-driven sampling methodology for HIV biological and behavioral surveillance: field experiences in international settings. AIDS Behav.

[11] Ministério da Saúde (BR), Secretaria ade Vigilância em Saúde, Programa Nacional de DST e Aids (2004). Avaliação da efetividade das ações de prevenção dirigidas às profissionais do sexo, em três regiões brasileiras.

[12] Salganik MJ (2006). Variance estimation, design effects, and sample size calculations for respondent-driven sampling. J Urban Health.

[13] Broutet N, Queiroz-Sousa A, Basilio FP, Sá HL, Simon F, Dabis F (1996). Prevalence of HIV-1, HIV-2 and HTLV antibody, in Fortaleza, Ceara, Brazil, 1993-1994. Int J STD AIDS.

[14] Macena RHM (2009). Profissionais do sexo feminino em três áreas do Ceará: fatores que ampliam a vulnerabilidade para DST/AIDS.

[15] Gökengin D, Geretti AM, Begovac J, Palfreeman A, Stevanovic M, Tarasenko O (2014). 2014 European guideline on HIV testing. Int J STD AIDS.

[16] Sousa RMRB (2013). “Não sei direito como é, eu só sei que é AIDS”: incentivos e barreiras ao teste de HIV/AIDS entre profissionais do sexo.

[17] Ministério da Saúde (BR), Secretaria de Vigilância em Saúde, Departamento de DST Aids e Hepatites Virais (2011). Pesquisa de Conhecimento Atitudes e Práticas na População Brasileira - PCAP.

[18] Silveira MF, Teixeira AMFB, Stephan LS, Rosenthal RM, Alves CL, Brum VMA (2009). Conhecimento sobre sorologia para sífilis e HIV entre profissionais do sexo de Pelotas, Brasil. DST J Bras Doenças Sex Transm.

[19] Aho J, Nguyen VK, Diakité S, Sow A, Koushik A, Rashed S (2012). High acceptability of HIV voluntary counselling and testing among female sex workers: impact of individual and social factors. HIV Med.

[20] Arora P, Nagelkerke NJ, Moineddin R, Bhattacharya M, Jha P (2013). Female sex work interventions and changes in HIV and syphilis infection risks from 2003 to 2008 in India: a repeated cross-sectional study. BMJ Open.

[21] Musheke M, Ntalasha H, Gari S, Mckenzie O, Bond V, Martin-Hilber A (2013). A systematic review of qualitative findings on factors enabling and deterring uptake of HIV testing in Sub-Saharan Africa. BMC Public Health.

[22] Ahoyo AB, Alary M, Ndour M, Labbé AC, Ahoussinou C (2009). VIH et infections sexuellement transmissibles chez les travailleuses du sexe au Benin. Med Trop (Mars).

[23] Beattie TS, Bhattacharjee P, Suresh M, Isac S, Ramesh B, Moses S (2012). Personal, interpersonal and structural challenges to accessing HIV testing, treatment and care services among female sex workers, men who have sex with men and transgenders in Karnataka state, South India. J Epidemio Community Health.

[24] Surratt HL, O’Grady CL, Kurtz SP, Buttram ME, Levi-Minzi MA (2014). HIV testing and engagement in care among highly vulnerable female sex workers: implications for treatment as prevention models. J Health Care Poor Underserved.

